# High Salinity Induces Different Oxidative Stress and Antioxidant Responses in Maize Seedlings Organs

**DOI:** 10.3389/fpls.2016.00276

**Published:** 2016-03-08

**Authors:** Hamada AbdElgawad, Gaurav Zinta, Momtaz M. Hegab, Renu Pandey, Han Asard, Walid Abuelsoud

**Affiliations:** ^1^Integrated Molecular Plant Physiology Research, Department of Biology, University of AntwerpAntwerp, Belgium; ^2^Department of Botany, Faculty of Science, University of Beni-SuefBeni-Suef, Egypt; ^3^Centre of Excellence Plant and Vegetation Ecology, Department of Biology, University of AntwerpAntwerp, Belgium; ^4^Division of Plant Physiology, Indian Agricultural Research InstituteNew Delhi, India; ^5^Department of Botany and Microbiology, Faculty of Science, Cairo UniversityGiza, Egypt

**Keywords:** salinity, maize, biomass, reactive oxygen species (ROS), oxidative stress, antioxidants

## Abstract

Salinity negatively affects plant growth and causes significant crop yield losses world-wide. Maize is an economically important cereal crop affected by high salinity. In this study, maize seedlings were subjected to 75 mM and 150 mM NaCl, to emulate high soil salinity. Roots, mature leaves (basal leaf-pair 1,2) and young leaves (distal leaf-pair 3,4) were harvested after 3 weeks of sowing. Roots showed the highest reduction in biomass, followed by mature and young leaves in the salt-stressed plants. Concomitant with the pattern of growth reduction, roots accumulated the highest levels of Na^+^ followed by mature and young leaves. High salinity induced oxidative stress in the roots and mature leaves, but to a lesser extent in younger leaves. The younger leaves showed increased electrolyte leakage (EL), malondialdehyde (MDA), and hydrogen peroxide (H_2_O_2_) concentrations only at 150 mM NaCl. Total antioxidant capacity (TAC) and polyphenol content increased with the increase in salinity levels in roots and mature leaves, but showed no changes in the young leaves. Under salinity stress, reduced ascorbate (ASC) and glutathione (GSH) content increased in roots, while total tocopherol levels increased specifically in the shoot tissues. Similarly, redox changes estimated by the ratio of redox couples (ASC/total ascorbate and GSH/total glutathione) showed significant decreases in the roots. Activities of enzymatic antioxidants, catalase (CAT, EC 1.11.1.6) and dehydroascorbate reductase (DHAR, EC 1.8.5.1), increased in all organs of salt-treated plants, while superoxide dismutase (SOD, EC 1.15.1.1), ascorbate peroxidase (APX, EC 1.11.1.11), glutathione-*s*-transferase (GST, EC 2.5.1.18) and glutathione reductase (GR, EC 1.6.4.2) increased specifically in the roots. Overall, these results suggest that Na^+^ is retained and detoxified mainly in roots, and less stress impact is observed in mature and younger leaves. This study also indicates a possible role of ROS in the systemic signaling from roots to leaves, allowing leaves to activate their defense mechanisms for better protection against salt stress.

## Introduction

Soil salinization is a serious threat to crop productivity and predicted to increase in the face of global climate change (FAO, 2011)^1^. Estimations show that as much as 12 billion US$ will be lost globally, each year, due to the reduction in agricultural production from salt-affected areas (Qadir et al., 2008; Flowers et al., 2010). The situation is worst in arid and semi-arid regions, which are deficient in water and face high temperatures, resulting in more water-loss from plants due to higher evapotranspiration rates, aggravating the effects of salinity.

Salinity stress induces a multitude of responses in plants including morphological, physiological, biochemical, and molecular changes (Ambede et al., 2012; Abreu et al., 2013). It causes ionic imbalance, which results in ionic toxicity, osmotic stress, and generation of reactive oxygen species (ROS; Chaparzadeh et al., 2004; Chawla et al., 2013). For instance, accumulation of Na^+^ under salinity stress competes with K^+^ binding in proteins, causing inhibition of protein synthesis and metabolic enzymes (Schachtman and Liu, 1999; Pardo and Quintero, 2002). High concentrations of NaCl outside the roots reduce the water potential, making it more difficult for plants to extract water, and results in osmotic stress. In leaves, high salt levels cause stomatal closure, impairment of electron transport and the photosynthetic apparatus, leading to reduced photosynthesis and productivity (Abreu et al., 2013; Deinlein et al., 2014). To cope with osmotic stress, salt-stressed plants tend to accumulate compatible solutes such as proline (Pro) and glycine betaine (Holmström et al., 2000; Qureshi et al., 2013), that decrease the cytoplasmic osmotic potential, enabling water absorption (Pottosin et al., 2014; Puniran-Hartley et al., 2014).

High salinity also induces the formation of ROS within plant cells, and its over accumulation results in oxidative damage of membrane lipids, proteins and nucleic acids (Pérez-López et al., 2009; Gill and Tuteja, 2010). To scavenge high ROS levels, an efficient system of non-enzymatic and enzymatic antioxidants is involved (Apel and Hirt, 2004; Gill and Tuteja, 2010; Karuppanapandian et al., 2011). Non-enzymatic antioxidants include phenolics, flavonoids, tocopherols, ASC, and GSH (Munné-Bosch, 2005; Rai et al., 2013; Gupta and Huang, 2014; Rakhmankulova et al., 2015; Talbi et al., 2015). Enzymatic antioxidants include superoxide dismutase (SOD), peroxidase (POX), catalase (CAT), as well as the enzymes of the ascorbate (ASC)–glutathione (GSH) cycle [GSH reductase (GR), ASC peroxidase (APX), monodehydroascorbate dehydrogenase (MDHAR), and dehydroascorbate reductase (DHAR)] that detoxify ROS (Gill and Tuteja, 2010; Foyer and Noctor, 2011; Chawla et al., 2013). Up-regulation of antioxidants has been observed in salt tolerant cultivars of tomato, pea, *Jatropha*, and *Calendula* (Hernandez et al., 2000; Chaparzadeh et al., 2004; Mittova et al., 2004; Gao et al., 2008), suggesting a pertinent role of antioxidants in alleviating salt stress-induced oxidative damage. So far, no salinity-specific ROS or salinity-specific antioxidants have been identified in plants in general, the same species of ROS are produced under different abiotic and some biotic stresses, in part as by products of the stress and to serve as signals to nearby tissues as well (reviewed by Apel and Hirt, 2004). However, some salinity-specific physiological and biochemical mechanisms are identified. For instance, the uptake, compartmentalization and/or excretion of ions from specialized cells under salinity stress is essential for normal growth (Gupta and Huang, 2014; Viehweger, 2014).

Roots are the first organs to undergo salinity stress, and show greater reduction in growth than shoots (Lazof and Bernstein, 1999). However, other plants, such as avocado, showed a higher root to shoot ratio under high salinity (Bernstein et al., 2004). Moreover, some developmental stages, including germination, seedling emergence, and flowering are more sensitive to salt stress, and therefore the effects of high salinity depend on the plant developmental stage (Houle et al., 2001). Interestingly, age-related differences in responses in plant cells, tissues and organs have been observed. For instance, mature maize leave cells (at distal leaf parts), are more sensitive to high salinity than younger cells (from actively expanding leaf parts) which have higher expression of genes of the antioxidant enzymes (Kravchik and Bernstein, 2013). Also, cotton and *Fragaria virginiana* leaves of different ages, under drought stress, showed different stomatal conductance and osmotic potential adjustment responses (Jordan et al., 1975). However, differences in oxidative stress and antioxidant defenses, in different organs and developmental stages, have been much less studied.

Maize is an economically important cereal crop and its productivity is affected by high soil salinity in various parts of the world. Therefore, this work aims to elucidate the effects of salinity on maize growth. Based on our previous observations, we hypothesized that different organs and/or leaf tissues of different developmental stages may respond differently to high salinity stress and trigger specific defense mechanisms. We therefore compared oxidative stress and antioxidant responses of roots, mature leaves (basal leaf-pair 1, 2) and young leaves (distal leaf-pair 3, 4) harvested from 3 weeks old, salt-treated and non-treated maize seedlings.

## Materials and Methods

### Plant Material and Growth Conditions

Maize (*Zea mays* L. cv Giza 119) seeds were obtained from Agricultural Research Center, Giza, Egypt. Giza 119 is a local commercial variety first developed in 1973. Seeds were soaked on filter paper saturated with distilled water, and incubated at 26°C in the dark. Three days later, seedlings selected for uniform growth were transplanted into sandy soil (90% sand, pH 7.6). The soil initially contained 1.2% carbon, 15 mg nitrate-nitrogen (N), 1 mg ammonium-N, 10 mg phosphorus (P)/g air dry soil at a humidity of 0.30 g water/g dry soil. Growth conditions were; 16/8 h day/night photoperiod, 26/22°C temperature, and 70/80% relative humidity. High salinity treatments started immediately after transplantation by irrigating soils daily with 25 ml of 75 mM or 150 mM NaCl. These treatments were selected based on a preliminary study, where 75 mM and 150 mM showed mild and sever effects on plant growth, respectively. After 3 weeks of growth, roots, mature leaf-pair 1, 2 (L1,2) and distal leaf-pair 3, 4 (L3,4), were harvested. Samples were immediately frozen in liquid nitrogen and stored at -80°C, for further biochemical analyses. Dry weight (DW) of the plant organs was measured after drying the tissues for 5 days at 70°C.

### Na^+^, K^+^, and Cl^-^ Content Determination

Na^+^, K^+^, and Cl^-^ content was determined in 100 mg DW of plant material. Samples were digested in 5 ml of HNO_3_/H_2_O (5:1), and supernatants after clarifying the digestate were analyzed by mass spectrometry (ICP-MS, Finnigan Element XR, Scientific, Bremen, Germany) as described previously (Hamad et al., 2015).

### Oxidative Stress Markers

#### Malondialdehyde (MDA)

Malondialdehyde content was assayed according to Hodges et al. (1999). Fifty milligram fresh weight (FW) of tissue was homogenized in 1 ml of 80% (v/v) ethanol using a MagNA Lyser (Roche, Vilvoorde, Belgium). After centrifugation, the supernatant reacted with thiobarbituric acid to produce pinkish red chromogen, thiobarbituric acid-malondialdehyde (TBA-MDA). Absorbance was measured at 440, 532, and 600 nm by using a micro-plate reader (Synergy Mx, Biotek Instruments Inc., Vermont, VT, USA). MDA content was calculated and expressed as nmol/g FW tissue.

#### H_2_O_2_ Concentration

Fifty milligram fresh tissue was homogenized in 0.5 ml 0.1% TCA, centrifuged and 50 μl was used in the assay. Hydrogen peroxide (H_2_O_2_) concentration was measured according to Jiang et al. (1990), based on the peroxide-mediated oxidation of Fe^2+^, followed by reaction of Fe^3+^, with xylenol orange. Specificity for H_2_O_2_ was tested by eliminating H_2_O_2_ from the reaction mixture with CAT.

#### Electrolyte Leakage

Membrane leakage of cells was measured by cutting 1 cm^2^ disks of leaf tissues and segments of root tissues, washing thoroughly, and incubation in 20 ml deionized water for 18 h at room temperature. After this incubation leaf disks were boiled for 30 min. The conductivity of the incubation solutions was measured at three time points using a conductivity meter (WTW GmbH, Weilheim, Germany; Lutts et al., 1995).

### Antioxidant Molecules

#### Total Antioxidant Capacity (TAC)

Two hundred milligram (FW) frozen plant tissue was ground with mortar and pestle in liquid nitrogen, and antioxidants were extracted in 2 ml of ice-cold 80% ethanol. FRAP (ferric reducing antioxidant power) reagent (300 mM acetate buffer (pH 3.6), 0.01 mM 2,4,6-tripirydylo-*S*-triazine (TPTZ)) in 0.04 mM HCl and 20 mM FeCl_3_.6H_2_O), was mixed with the extract and measured at 600 nm using a microplate reader (Benzie and Strain, 1999). Trolox was used as a standard.

#### Polyphenols and Flavonoids

Polyphenols and flavonoids were extracted by homogenizing 50 mg tissues in 0.5 ml 80% ethanol (v/v), centrifuged, pellet was washed twice each with 0.5 ml 80% ethanol (v/v), supernatants were pooled. Total phenolic content was determined using a Folin–Ciocalteu assay according to Zhang et al. (2006). Gallic acid was used as a standard. Flavonoid content was estimated using the modified aluminum chloride calorimetric method (Chang et al., 2002), with quercetin as a standard.

#### Ascorbate and Glutathione

One hundred milligram (FW) of frozen tissues were ground in a Mag NALyser (Roche, Vilvoorde, Belgium), and extracted in ice-cold 6% (v/v) phosphoric acid. Reduced ASC and GSH contents were determined by HPLC analysis (Potters et al., 2004). The identity of the peaks was confirmed using an in-line diode array detector (DAD, SPD-M10AVP, Shimadzu). Total ASC and GSH concentration was determined after reducing the samples with 40 mM DTT and the ASC and GSH redox status are calculated as the ratio between reduced and total amount of ASC and GSH, respectively.

#### Tocopherols

One hundred milligram (FW) of frozen plant tissue was extracted in 6 ml of hexane, and centrifuged at 14,000 × *g* for 15 min. Extracts were dried (CentriVap concentrator, Labconco, Kansas City, MO, USA) and resuspended again in hexane. Tocopherols were separated and quantified by HPLC analysis (Shimadzu, Hertogenbosch, The Netherlands, normal phase conditions, Particil Pac 5 mm column material, length 250 mm, i.d. 4.6 mm). Dimethyl tocol (DMT) was used as internal standard (5 ppm). Data were analyzed with Shimadzu Class VP 6.14 software.

### Enzyme Activities

Superoxide dismutase, POX (EC 1.11.1.7), CAT, GSH POX (GPX), APX, GR, glutathione-*S*-transferase (GST), monodehydroascorbate reductase (MDHAR, EC 1.6.5.4) and DHAR were determined in an homogenate of 100 mg (FW) of leaf tissues, prepared in 1 ml of 50 mM potassium phosphate buffer (pH 7.0), containing 10% (w/v) polyvinylpyrrolidone (PVP), 0.25% (v/v) Triton X-100, 1 mM phenylmethylsulfonyl fluoride(PMSF) and 1 mM ASC, by using a MagNA Lyser (Roche, Vilvoorde, Belgium). SOD activity was determined according to Dhindsa et al. (1982) by measuring the inhibition of NBT (nitroblue tetrazolium) reduction at 560 nm. POX activity was determined by the oxidation of pyrogallol (ε_430_ = 2.47 mM^-1^ cm^-1^; Kumar and Khan, 1982). CAT activity was assayed according to the Aebi (1984) by monitoring the decomposition of H_2_O_2_ at 240 nm. APX, MDHAR, DHAR, and GR activities were measured by the methods of (Murshed et al., 2008). GST activity was estimated by measuring the conjugation of GSH with excess 1-chloro-2,4-dinitrobenzene (CDNB) at 340 nm (ε_340_ = 0.0096 μM^-1^ cm^-1^; Habig et al., 1974). GPX activity was assayed by measuring the decrease in NADPH absorbance measured at 340 nm (ε_340_ = 6.22 mM^-1^cm^-1^; Drotar et al., 1985). All activity measurements were scaled down for semi-high throughput using a micro-plate reader (Synergy Mx, Biotek Instruments Inc., Winooski, VT, USA), and optimized to obtain linear time and protein concentration dependence.

### Protein Concentration

The soluble protein was extracted by homogenizing 100 mg fresh tissue in content was estimated according to the method of Lowry et al. (1951).

### Proline concentration

Proline content was estimated by homogenizing 0.2 g fresh weight in 2 ml of 3% aqueous sulfosalicylic acid, centrifugation at 10,000 rpm for 30 min. The supernatant was decanted and pellet was washed twice with 3% aqueous sulfosalicylic acid. The supernatants were pooled and proline content was measured using ninhydrin reagent and toluene extraction (Bates et al., 1973)

### Statistical Analysis

All results were analyzed by one-way ANOVA using SPSS 16.0 statistical software and significant differences between the means of parameters (*n* = 8) were determined by using the Duncan test (*P* < 0.05). The treatments of each organ were compared with their corresponding control where significant differences were given different letters.

## Results

### Biomass

**FIGURE 1:**
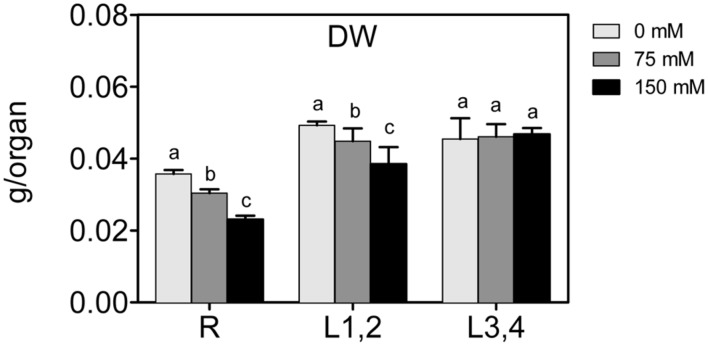
**Effect of different salinity levels on dry weight (DW) of root (R), mature leaf pair (L1,2), and young leaf pair (L3,4) of 3 weeks old maize seedlings.** Values are means of at least three replicates and significant differences between means, as determined by Duncan test (*P* < 0.05), are indicated by different letters.

To assess the effect of high salinity (NaCl) on maize growth, biomass (DW) of roots, L1,2, and L3,4, was measured. Increase in the NaCl concentration resulted in a significant decrease in the biomass of roots and L1,2, and larger decline was observed at the higher (150 mM) NaCl concentration (**Figure 1**). However, L3,4 harvested from NaCl-treated plants showed no statistical difference in the biomass compared to the controls. This suggests that severe salinity reduced growth progressively from roots along the vertical gradient of the maize plant, with the root found to be most sensitive (-40%), followed by L1,2 (-20%).

### Na^+^, K^+^, and Cl^-^ Content

**FIGURE 2:**
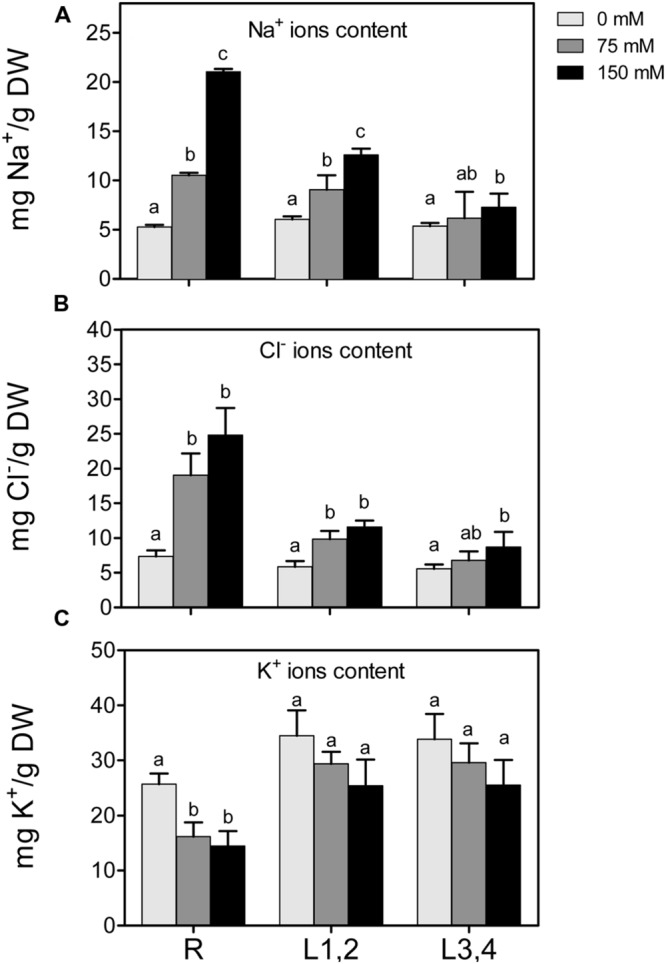
**Effect of different salinity levels on Na^+^, Cl^-^, and K^+^ ion content of root (R), mature leaf pair (L1,2), and young leaf pair (L3,4) of 3 weeks old maize seedlings.** Values are means of at least three replicates and significant differences between means, as determined by Duncan test (*P* < 0.05), are indicated by different letters.

High soil salinity causes over-accumulation of Na^+^ inside the cell, which may result in ionic imbalances and metabolic toxicity. Na^+^ content increased significantly with the increase of applied NaCl concentration in root and L1,2 but Na^+^ accumulation was significant in L3,4 only under 75 mM treatment and not significant when further increased to 150 mM (**Figure 2A**). As with the effect of salt on biomass, there was an apparent gradient, with highest Na^+^ accumulation in roots (+404%), followed by L1,2 (+208%) and L3,4 (+137.3%), under severe salinity stress, compared to their respective controls. The change in Cl^-^ content of different organs under salinity behaved similar to Na^+^ but the L1,2 content of Cl^-^ increased with salinity in a concentration independent manner (**Figure 2B**). K^+^ content of roots decreased with the increase in salinity stress but in L1,2 and L3,4, no significant change was observed (**Figure 2C**).

### Oxidative Stress Markers

**FIGURE 3:**
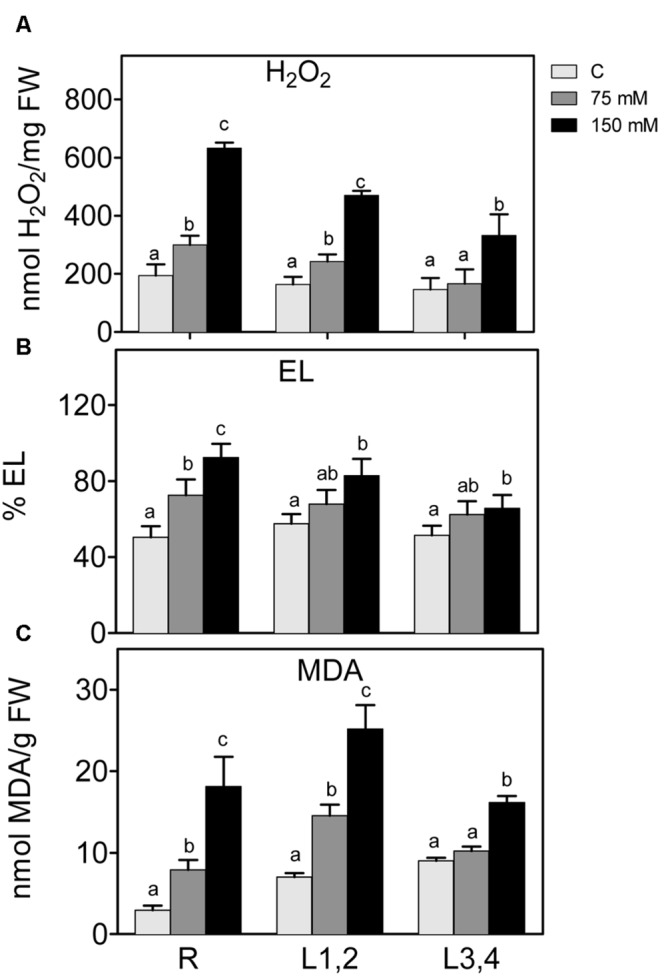
**Effect of different salinity levels on H_2_O_2_**(A)** relative electrolyte leakage (EL, **B**), and lipid peroxidation measured as malondialdehyde content **(C)** of root (R), mature leaf pair (L1,2), and young leaf pair (L3,4) of 3 weeks old maize seedlings.** Values are means of at least three replicates and significant differences between means, as determined by Duncan test (*P* < 0.05), are indicated by different letters.

Salt stress also leads to higher accumulation of ROS, which disturb cellular redox homeostasis, and result in oxidative damage. H_2_O_2_ content and markers of oxidative damage of cell membranes, i.e., electrolyte leakage (EL) and lipid peroxidation (MDA) were measured (**Figure 3**). Increase in the NaCl concentration resulted in a significant increase in the accumulation of H_2_O_2_ content in roots and L1,2. However, in L3,4, the increase was only observed at the highest NaCl (150 mM) concentration. Following the trend of H_2_O_2_, EL, and MDA also increased significantly (*p* < 0.05) in root and L1,2 but only at 150 mM NaCl in L3,4. Thus, oxidative stress parameters indicate that root tissues suffered the most from the salinity-induced oxidative stress, followed by L1,2 and L3,4 was the least affected.

### Antioxidant Defense Systems

#### Non-enzymatic Antioxidants

**FIGURE 4:**
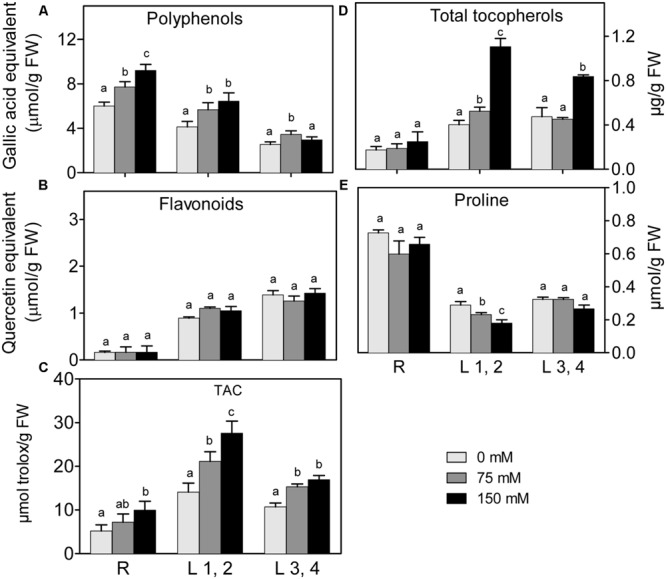
**Effect of different salinity levels on polyphenols **(A)**, flavonoids **(B)**, total antioxidant capacity (TAC, **C**), total tocopherols **(D)**, and Proline **(E)** content of root (R), mature leaf pair (L1,2) and young leaf pair (L3,4) of 3 weeks old maize seedlings.** Values are means of at least three replicates and significant differences between means, as determined by Duncan test (*P* < 0.05), are indicated by different letters.

Changes in polyphenol and flavonoid content in roots, L1,2 and L3,4 under 75 mM and 150 mM NaCl, are shown in (**Figures 4A,B**). Polyphenol content of roots, increased with increasing salinity in a concentration dependent manner. However, its increment in L1,2 and L3,4 under salinity stress was concentration independent. High salinity did not affect levels of flavonoids in any of the tested organs. Increasing severity of salinity induced increases in the TAC, as determined by the FRAP-assay (**Figure 4C**), of L1,2 in a concentration-dependent manner. Root and L3,4 tissues were less responsive, their antioxidant capacity only slightly increased under mild or severe salinity stress. Slight increase in total tocopherol content was observed in L1,2 under mild salinity stress but not in root or L3,4 tissues. However, under severe salinity stress, the total tocopherol content increased significantly only in L1,2 and L3,4 (**Figure 4D**). The Pro content slightly decreased in L1,2 leaves, but did not show significant changes in maize root and L3,4 leaves (**Figure 4E**) under exposure to salinity stress.

#### Enzymatic Antioxidants and ASC/GSH-Cycle Components

**FIGURE 5:**
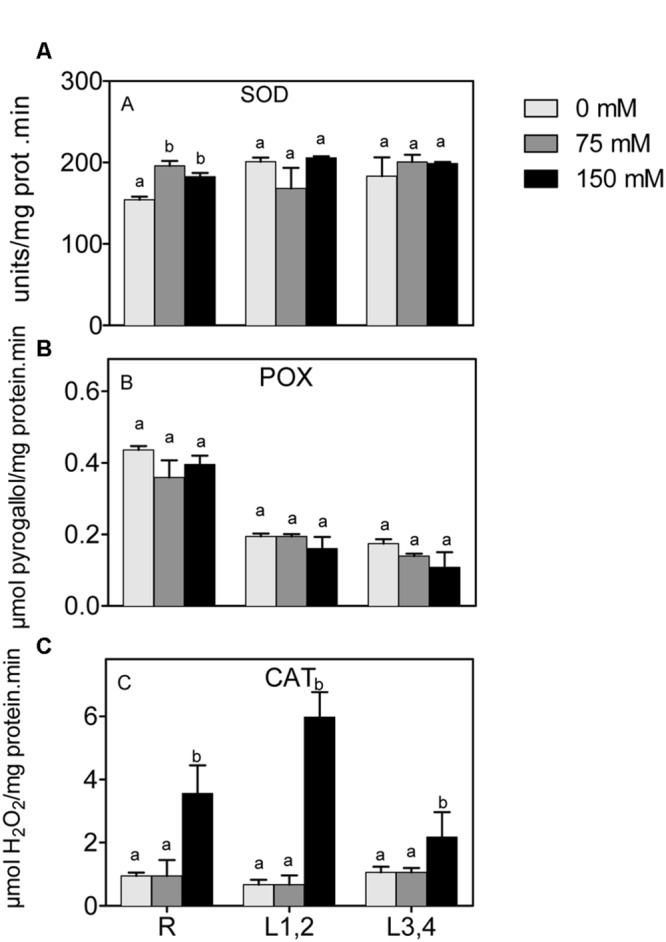
**Effect of different salinity levels on superoxide dismutase **(A)**, peroxidase **(B)**, and catalase **(C)** activities of root (R), mature leaf pair (L1,2), and young leaf pair (L3,4) of 3 weeks old maize seedlings.** Values are means of at least three replicates and significant differences between means, as determined by Duncan test (*P* < 0.05), are indicated by different letters.

Mild or severe salinity stress increased SOD activity in root in a concentration independent manner. L1,2 and L3,4 content of SOD activity was not significantly affected by salinity stress (**Figure 5A**). POX did not show significant differences in activity upon exposure to mild or severe salinity in either roots, L1,2 or L3,4 leaves (**Figure 5B**). Only the highest salinity level resulted in a significant increase in CAT activity, and this was strongest in roots and L1,2 leaves (**Figure 5C**).

**FIGURE 6:**
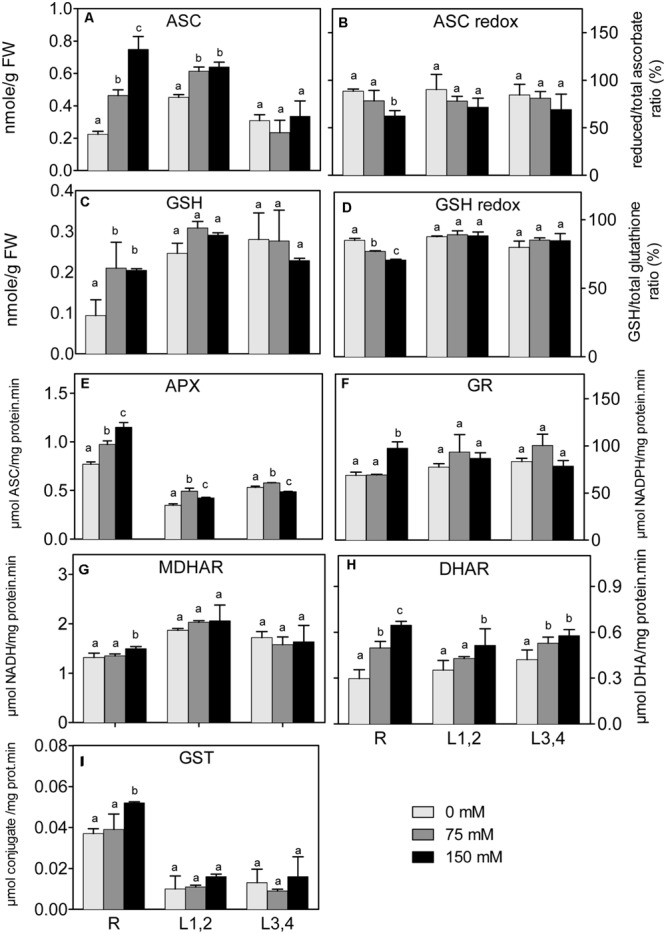
**Effect of different salinity levels on reduced ascorbic acid (ASC, **A**), ascorbate redox status (reduced/total ascorbate, **B)**, reduced glutathione (GSH, **C**), glutathione redox status (reduced/total glutathione ratio, **D)**, ascorbate peroxidase (APX, **E**), glutathione reductase (GR, **F**), monodehydroascorbate reductase (MDHAR, **G**), dehydroascorbate reductase (DHAR, **H**), and glutathione-*S*-transferase (GST, **I**) activities of root (R), mature leaf pair (L1,2), and young leaf pair (L3,4) of 3 weeks old maize seedlings.** Values are means of at least three replicates and significant differences between means, as determined by Duncan test (*P* < 0.05), are indicated by different letters.

In all plant parts, except L3,4, there was a significant increase of ASC under salinity stress (**Figure 6A**). However, the ascorbate redox status (ASC/total ascorbate, **Figure 6B**) decreased in root upon the increase in salinity. The L1,2 and L3,4 GSH content, and the GSH redox status (GSH/total glutathione), did not significantly change under any degree of salinity stress (**Figures 6C,D**). The, root GSH content increased, but the redox status decreased under salinity. APX activity (**Figure 6E**) in roots increased progressively with the increase in salinity strength, whereas in L1,2 and L3,4 tissues, its activity increased only slightly under mild but not severe salinity (**Figure 6E**). GR, MDHAR, and GST activities increased in root tissues only under severe salinity. Their activities in L1,2 and L3,4 was not affected by salinity (**Figures 6F,G,I**). DHAR activity (**Figure 6H**) in root tissues increased with increase in salinity in a concentration dependent manner. Only severe salinity increased DHAR activity in L1,2 and L3,4.

## Discussion

Soil salinity has emerged as a serious problem affecting crop productivity as well as distribution and survival of wild plants (Shabala et al., 2014). To contribute to our understanding of the mechanisms underlying salinity stress responses, we exposed maize seedlings to two different salinity levels, and compared responses in roots, mature leaves (L1,2) and young distal leaves (L3,4).

The results show that biomass (DW) of roots and older leaves (L1,2) decreased linearly with the increase in salinity, but biomass of young leaves (L3,4) was not affected by any level of salinity. This result may indicate lowered growth in roots and lowered CO_2_ assimilation in L1,2 but not L3,4 tissues was correlated to salinity. Growth inhibition in these organs also correlates linearly with increasing Na^+^ accumulation within tissues. Na^+^ and Cl^-^ ions accumulation in leaf tissues could have resulted in stomatal closure and/or damage of photosynthetic machinery that in turn resulted in lower CO_2_ assimilation and lower growth and DW accumulation. A correlation between Na^+^ ion accumulation in root and shoot tissues and lowered DW was also previously observed. Pumpkin (*Cucurbita pepo*) and ground nut (*Vigna subterranea*) root and shoot length, FW and DW decreased in correlation to increasing salinity (Ambede et al., 2012; Kurum et al., 2013). Moreover, a decrease in chlorophyll content and photosynthetic efficiency under salinity stresses was observed in various plant species (Rout et al., 1997; Jamil et al., 2007; Ambede et al., 2012). Na^+^ ions accumulated in the roots of salt tolerant as well as salt sensitive green bean seedlings upon exposure to salinity stress. However, Na^+^ accumulated in the young leaves of salt sensitive genotype but in the older leaves of salt tolerant genotype (Yasar et al., 2006). The accumulation of Na^+^ in roots caused nutrient imbalance in the root tissues as indicated by decreased K^+^/Na^+^ ratio with the increase in salinity. The nutrient imbalance, in turn, contributed to lower growth and dry matter accumulation in root tissues. Similar results in maize has been found in salt sensitive maize (Hussain et al., 2014).

The accumulation of Na^+^ ions in roots and L1,2 was accompanied by increased levels of oxidative stress parameters, i.e., EL, MDA, and H_2_O_2_, as well as decreased GSH and ASC redox status in root tissues. High salinity has been reported to induce oxidative stress in different plants and tissues (Ashraf and Harris, 2004; Chawla et al., 2013). Under salinity stress, the level of ROS increases in the plant tissues as a result of irregularities in the electron transport chain and accumulation of photoreducing power. This excess of electrochemical energy can be dissipated through the Mehler reaction, resulting in ROS generation, including H_2_O_2_ (Asada, 1999), and damage of membranes, reflected in elevated EL and MDA levels (Pérez-López et al., 2009; Sharma et al., 2012; Chawla et al., 2013).

The results show that oxidative stress defense in maize under high salinity occurs through non-enzymatic and enzymatic mechanisms. Although there are common responses to salinity stress in different organs (e.g., CAT, DHAR, APX), there are also organ-specific reactions. Roots and older leaves (L1,2) responded by increasing polyphenol content and ASC, as non-enzymatic antioxidants, correlating with higher Na^+^ accumulation in these tissues. The accumulation of tocopherol in L1,2 and L3,4 but not in root tissues under salinity stress could be due to the specificity of tocopherols in scavenging singlet oxygen radicals in photosystem II (Kruk et al., 2005). Similarly, Hichem et al. (2009) found that salt-challenged maize plants accumulated higher polyphenols, but young leaves accumulated more polyphenols than older ones. ASC and tocopherols content have been shown to increase in leaves of tomato to protect them against oxidative stress under triazole treatment, as well as in wheat leaves under high salinity (Salama et al., 1994; Tuna, 2014). On the other hand, levels of ASC and tocopherols declined in de-etiolated rice leaves under salinity stress (Turan and Tripathy, 2013), these conflicting results could be due to plant/tissue specificity. At the level of organ specificity, our results also showed increased GSH level in response to high salinity that was root-specific. This could be ascribed to the increased demand and metabolism of sulfur under stress for biosynthesis of antioxidants such as GSH (Gill et al., 2013). Moreover, the root-located biosynthesis of the stress hormone ABA, which may act as a stress signal to the shoot (Bittner et al., 2001), increases under different abiotic stresses and requires sulfur supply. Upregulated expression of the sulfate transporter gene, *AtSULTR3;1*, and higher sulfate absorption are observed in salt-stressed *Arabidopsis* plants (Cao et al., 2014; Gallardo et al., 2014). The increased absorption of sulfate affects ABA and GSH levels in tissues under high salinity (Ruiz and Blumwald, 2002; Gill et al., 2013; Cao et al., 2014) and under drought stress (Chan et al., 2013; Gill et al., 2013). The increased content of ASC and GSH, accompanied by the reduced ASC and GSH redox status, indicates the crucial role of the ASC–GSH cycle for scavenging ROS especially in root tissues of maize seedlings. Similarly, Hernandez et al. (2000) have shown that the salinity tolerant, but not the sensitive variety, accumulated higher activities and transcripts of the ASC–GSH cycle.

At the level of enzymatic oxidative stress defenses, some responses to high salt appear to occur more general in roots and shoots (CAT, APX, and DHAR induction), whereas root-specific antioxidant enzymes (i.e., SOD, GR, and MDHAR) has been found. Yet other antioxidant enzymes, POX and MDHAR, were not affected by salinity stress in any of the tested organs. CAT, APX, and DHAR activities have been previously reported as general enzymatic antioxidant enzymes in different plant tissues and species, such as barley and rice (Pérez-López et al., 2009; Chawla et al., 2013). CAT has been shown by Gao et al. (2008) to be a major enzymatic antioxidant in radicles of *Jatropha curcas L.* challenged with salinity, especially under moderate salinity levels of 50 to 100 mM NaCl, compared to hypocotyl and cotyledons. Similarly, Chaparzadeh et al. (2004) showed increased CAT activity in leaves of *Calendula offıcinalis* under NaCl concentrations up to 100 mM, while MDHAR activity did not increase in either leaves or roots under salinity stress. Moreover, under sulfur deficiency, maize seedlings showed an increase in CAT activity in leaf sheaths and blades and decreased activity in roots while SOD activity decreased in roots and leaf sheaths but not in blades compared to their controls (Chorianopoulou et al., 2012) and these differential changes where ascribed to the differential ROS production in different organs under sulfur deficiency. Leaves of salinity tolerant rice cultivars showed higher activities of SOD and GR (Chawla et al., 2013). On the contrary, leaves of *Calendula officinalis* have lower SOD activity under salinity but enhanced GR activity compared to their control (Chaparzadeh et al., 2004).

Taken together, our results indicate that roots have a wide array of antioxidant metabolites and enzymes, mainly of the ASC–GSH cycle, that are upregulated in response to salinity stress. Tocopherol, on the other hand appears a more shoot-specific antioxidant in maize seedlings. Other antioxidant components did not show particular organ-specific increases, e.g., CAT, SOD, DHAR, and APX.

A root-specific increase in GST activities was observed under severe salinity. This increment in GST activity may be to detoxify secondary products of oxidative stress such as 4-hydroxynonenal, a toxic alkenal released due to oxidative damage of membranes (Edwards et al., 2000). Although GST is not strictly an antioxidant enzyme, rather it supports the detoxification of endobiotics and xenobiotics. Overexpression of a Tau-class GST in transgenic *Arabidopsis* and tobacco plants improved their oxidative and drought tolerance (Yu et al., 2003; Sharma et al., 2014).

Interestingly, Pro content did not change very much under any level of salinity in any tested organs. This is in contrast to many previous reports where Pro levels were increased under salinity stress to decrease the cellular water potential and improve water uptake, and possibly scavenge ROS molecules (Patel and Vora, 1985; Verslues and Sharma, 2010; Soshinkova et al., 2013). However, similar to our results *Sorghum bicolor* leaves showed no significant change in Pro content under salinity stress while in *S. sudanense* Pro decreased slightly with salinity (Pinho de Oliveira et al., 2013). Moreover, different maize varieties differed in Pro accumulation under salinity stress. Variety K3615.1 did not accumulate significant amounts of Pro under 50–100 mM NaCl, while K3653.2 showed decreased Pro content under elevated salinity, and other varieties accumulated large amounts of Pro upon NaCl treatment (Molazem and Bashirzadeh, 2015). The unchanged Pro levels under salinity stress in our study, could be due to the simultaneous upregulation of Pro synthesis and catabolizing enzymes (e.g., proline dehydrogenase) as a feedback response. Soshinkova et al. (2013) showed that upon addition of proline to the outer medium of cell suspensions, or to the nutrient solution of *Thellungiella salsuginea*, resulted in activation of proline dehydrogenase. It could also be ascribed to different thresholds of different maize varieties to synthesize proline under salinity stress. Moreover, the activation of enzymatic and non-enzymatic organic osmolytes/antioxidant defense molecules such as glycine betaine, ASC, GSH, SOD, CAT, etc. under salinity could be an alternative to proline to alleviate salt-induced damages.

These results indicate although there is universal antioxidant defense mechanisms apply for all tissues and developmental stages of the same plant. However, out of the arsenal of alternative antioxidant mechanisms available in all maize tissues, each organ uses mechanisms that are most efficient and in harmony with the other metabolic networks within that specific organ at the particular point of its age, to cope with the salinity and the resulting oxidative stress. Deciphering these specific alternatives could help developing more efficient metabolic engineering mechanisms specific to different organs and ages to cope with specific stress conditions. For example, engineering maize plants (var. Giza 119) with higher level of expression of key enzymes of ASC–GSH cycle in root, and synthesizing higher levels of tocopherols in shoots, and/or plants with reduced transport of Na^+^ from root to shoot, might help to cope with salinity stress at least at the seedling stage of the plant life.

## Author Contributions

All authors listed, have made substantial, direct and intellectual contribution to the work, and approved it for publication.

## Conflict of Interest Statement

The authors declare that the research was conducted in the absence of any commercial or financial relationships that could be construed as a potential conflict of interest.

## Footnotes

1 http://faostat.fao.org/site/339/default.aspx
